# Supplementary material for: Morphology of the Antennal Sensilla of the Nymphal Instars and Adults in Notobitus meleagris (Hemiptera: Heteroptera: Coreidae)
Source: Insects. 2023 Apr 1;14(4):351. doi: 10.3390/insects14040351 (PMC10143563; doi:10.3390/insects14040351)
Supplement: Supplementary file 1 [file insects-14-00351-s001.zip › insects-2309096-supplementary.pdf]

Table S1: Length of the antennae of each segment of *N. meleagris*.

Instar	Sc（μm）		Pe（μm）		F1（μm）		F2（μm）		Total (μm)
	**Length**	**Diameter**	**Length**	**Diameter**	**Length**	**Diameter**	**Length**	**Diameter**	**Length**
1	647.60±47.53 e	103.96±5.96 d	584.04±42.77 e	84.23±1.94 d	714.90±38.35 f	91.45±6.33 d	1523.40±26.41 e	103.03±9.26 c	3469.94±47.08 f
2	1012.60±83.58 e	116.12±6.69 d	1147.00±28.36 d	91.78±11.37 cd	1205.00±16.80 e	96.18±6.92 d	2147.40±17.24 de	127.83±7.34 c	5512.00±102.07 e
3	1575.60±68.50 d	177.08±7.08 c	1517.20±87.61 d	129.15±6.26 c	1682.80±59.10 d	140.11±6.79 c	2795.40±75.43 d	153.66±4.90 bc	7571.00±260.02 d
4	2268.80±68.96 c	250.23±6.77 b	2237.60±35.72 c	184.74±10.34 b	2277.00±44.71 c	181.03±6.25 b	3773.00±55.13 c	183.00±10.64 b	10556.40±184.16 c
5	3052.00±114.81 b	341.25±11.14 a	3029.20±47.65 b	257.67±7.03 a	2942.00±55.62 b	251.59±10.01 a	4550.40±186.39 b	264.15±21.52 a	13573.60±307.31 b
M	3516.60±182.34 a	371.60±18.20 a	3345.00±271.37 ab	266.73±12.13 a	3403.00±155.66 a	265.54±5.64 a	5459.80±323.71 ab	246.48±9.64 a	15724.40±863.49 a
F	3531.00±90.72 a	359.70±11.59 a	3578.00±57.65 a	272.80±6.54 a	3434.00±73.39 a	265.80±8.34 a	5185.60±145.54 a	251.50±13.03 a	15728.60±338.90 a
Degree freedom	(6, 28)	(6, 28)	(6, 28)	(6, 28)	(6, 28)	(6, 28)	(6, 28)	(6, 28)	(6, 28)
F value	134,232	121.221	103.860	93.686	203.818	112.438	95.616	29.399	160.089

Each column of data is analyzed together, and the letters in the table indicate significant differences ($p < 0.05$, Tukey).

Table S2: Morphological characteristics of antennae sensilla in nymphs of *N. meleagris*.

Type /subtypes of sensilla	Instar	Length	Degree freedom	F value/T value	Diameter	Degree freedom	F value/T value	Shape	Socket	Surface	Tip	Expected function
	1	31.736±1.293 c			2.188±0.120 b			Curved	No socket	Smooth	Blunt	
	2	47.369±1.060 b			2.298±0.108 b			Curved	No socket	Smooth	Blunt	Mechano-
St.1	3	48.189±1.093 b	(4, 165)	82.789	2.244±0.882 b	(4, 165)	7.734	Curved	No socket	Smooth	Blunt	and chemo-
	4	57.619±1.477 a			2.598±0.149 ab			Curved	No socket	Smooth	Blunt	reception
	5	58.607±0.941 a			3.031±0.153 a			Curved	No socket	Smooth	Blunt	
	1	-			-			-	-	-	-	
	2	-			-			-	-	-	-	Mechano-
St.2	3	-	6	-3.219	-	6	0.158	-	-	-	-	and chemo-
	4	29.973±2.825 b			3.648±0.878 a			Straight	Socket	Smooth	Blunt	reception
	5	40.153±1.422 a			3.488±0.504 a			Straight	Socket	Smooth	Blunt	
	1	-			-			-	-	-	-	
	2	-			-			-	-	-	-	Mechano-
St.3	3	-	38	-0.940	-	38	0.676	-	-	-	-	and chemo-
	4	61.294±2.066 a			4.787±0.330 a			Straight	Socket	Smooth	Blunt and shrinkage	reception
	5	65.398±3.846 a			4.425±0.421 a			Straight	Socket	Smooth	Blunt and shrinkage	
	1	7.122±0.193 d			1.476±0.261 d			Straight	No socket	Longitudinal grooved	Blunt and apertured	
	2	8.708±0.158 c			1.936±0.100 cd			Straight	No socket	Longitudinal grooved	Blunt and apertured	
Sb.1	3	9.593±0.566 bc	(4, 50)	28.940	3.076±0.189 ab	(4, 50)	20.753	Straight	No socket	Longitudinal grooved	Blunt and apertured	Chemo-reception
	4	10.532±0.338 b			2.458±0.145 bc			Straight	No socket	Longitudinal grooved	Blunt and apertured	
	5	12.400±0.422 a			3.309±0.261 a			Straight	No socket	Longitudinal grooved	Blunt and apertured	

	1	-			-			-	-	-	-	
	2	-			-			-	-	-	-	
Sb.2	3	35.511±1.318 a	(2, 42)	1.889	4.105±0.113 b	(2, 42)	5.610	Straight	Socket	Smooth	Blunt	Chemo-reception
	4	38.643±1.365 a			4.591±0.142 ab			Straight	Socket	Smooth	Blunt	
	5	40.745±2.724 a			5.271±0.388 a			Straight	Socket	Smooth	Blunt	
	1	54.003±1.933 c			4.097±0.154 c			Firm	Socket	Longitudinal grooved	Sharp and shrinkage	
	2	73.085±2.208 bc			4.773±0.167 bc			Firm	Socket	Longitudinal grooved	Sharp and shrinkage	
Sc.1	3	88.105±3.985 b	(4, 185)	39.704	5.783±0.197 b	(4, 185)	29.937	Firm	Socket	Longitudinal grooved	Sharp and shrinkage	Mechano-reception
	4	133.196±7.881 a			5.504±0.226b			Firm	Socket	Longitudinal grooved	Sharp and shrinkage	
	5	145.150±10.290 a			8.001±0.473 a			Firm	Socket	Longitudinal grooved	Sharp and shrinkage	
	1	38.375±2.371 c			2.317±0.219 d			Firm	Socket	Longitudinal grooved	Blunt	
	2	37.656±2.893 c			2.883±0.207 cd			Firm	Socket	Longitudinal grooved	Blunt	
Sc.2	3	37.548±2.267 c	(4, 70)	31.858	3.632±0.182 bc	(4, 70)	36.722	Firm	Socket	Longitudinal grooved	Blunt	Mechano-reception
	4	51.060±2.853 b			4.659±0.348 b			Firm	Socket	Longitudinal grooved	Blunt	
	5	75.620±3.892 a			6.938±0.454 a			Firm	Socket	Longitudinal grooved	Blunt	
	1	3.304±0.111 b			1.586±0.139 b			Straight	Socket	Longitudinal grooved	Oval and apertured	
Sco.1	2	4.383±0.326 b	(4, 30)	7.710	1.679±0.085 b	(4, 30)	5.155	Straight	Socket	Longitudinal grooved	Oval and apertured	Thermo- and hygro-reception
	3	3.691±0.229 ab			1.670±0.115 b			Straight	Socket	Longitudinal grooved	Oval and apertured	

4	5.171±0.533 a	1.511±0.092 b	Straight	Socket	Longitudinal grooved	Oval and apertured
5	5.464±0.318 a	2.219±0.166 a	Straight	Socket	Longitudinal grooved	Oval and apertured

Each sensilla subtype of data is analyzed together, and the letters in the table indicate significant differences ($p < 0.05$, Tukey and Student's t-test).

Table S3: Morphological characteristics of antennae sensilla in adults of *N. meleagris*.

Type /subtypes of sensilla	Sex	Length（μm）	Degree freedom	T value	Diameter（μm）	Degree freedom	T value	Shape	Socket	Surface	Tip	Expected function
St.1	Male	66.991±0.560 a	268	-1.453	3.349±0.090 a	268	-0.631	Curved	No socket	Smooth	Blunt	Mechano- and chemo-reception
	Female	68.900±1.189 a			3.430±0.092 a			Curved	No socket	Smooth	Blunt	
St.2	Male	34.033±1.182 a	22	-0.420	3.040±0.308 a	22	1.839	Straight	Socket	Smooth	Blunt	Mechano- and chemo-reception
	Female	34.848±1.539 a			2.311±0.250 a			Straight	Socket	Smooth	Blunt	
St.3	Male	60.889±1.192 a	96	2.868	3.632±0.113 a	96	0.741	Straight	Socket	Smooth	Blunt and shrinkage	Mechano- and chemo-reception
	Female	54.931±1.701 b			3.516±0.108 a			Straight	Socket	Smooth	Blunt and shrinkage	
Sb.1	Male	13.800±0.148 a	220	5.844	3.126±0.065 a	220	-0.079	Straight	No socket	Longitudinal grooved	Blunt and apertured	Chemo-reception
	Female	12.693±0.118 b			3.133±0.056 a			Straight	No socket	Longitudinal grooved	Blunt and apertured	
Sb.2	Male	35.957±0.693 a	90	-1.645	7.276±0.227 a	90	-2.523	Straight	Socket	Smooth	Blunt	Chemo-reception
	Female	33.551±0.835 b			6.552±0.189 b			Straight	Socket	Smooth	Blunt	
Sb.3	Male	15.095±0.538 a	86	2.218	2.874±0.086 b	86	2.453	Straight	Socket	Smooth	Blunt	Chemo-reception

	Female	16.265±0.465 a			3.171±0.080 a			Straight	Socket	Smooth	Blunt	
Sc.1	Male	126.254±3.321 a	390	0.657	10.114±0.214 a	390	1.287	Firm	Socket	Longitudinal grooved	Sharp and shrinkage	Mechano-reception
	Female	123.309±3.010 a			9.702±0.239 a			Firm	Socket	Longitudinal grooved	Sharp and shrinkage	
Sc.2	Male	80.400±1.775 b	124	-2.358	7.646±0.209 a	124	0.666	Firm	Socket	Longitudinal grooved	Blunt	Mechano-reception
	Female	85.760±1.420 a			7.446±0.217 a			Firm	Socket	Longitudinal grooved	Blunt	
Sco.1	Male	6.364±0.138 a	148	4.712	2.385±0.070 a	148	2.681	Straight	Socket	Longitudinal grooved	Oval and apertured	Thermo- and hygro-reception
	Female	5.363±0.161 b			2.116±0.072 b			Straight	Socket	Longitudinal grooved	Oval and apertured	
Sco.2	Male	2.142±0.094 a	62	1.894	1.733±0.079 a	62	1.471	Embedded	Socket	Smooth	Oval	Thermo- and hygro-reception
	Female	1.887±0.096 a			1.551±0.096 a			Embedded	Socket	Smooth	Oval	
Sca	Male	10.110±0.411 a	38	-1.435	7.379±1.087 b	38	-3.126	Oval	Socket	Smooth	Oval	Mechano-reception
	Female	11.997±1.250 a			11.663±0.835 a			Oval	Socket	Smooth	Oval	

Each sensilla subtype of data is analyzed together, and the letters in the table indicate significant differences ($p < 0.05$, Student's t-test)
